# Application of Circular Patch Plasty (Dor Procedure) or Linear Repair Techniques in the Treatment of Left Ventricular Aneurysms

**DOI:** 10.21470/1678-9741-2017-0093

**Published:** 2018

**Authors:** Ugur Kaya, Abdurrahim Çolak, Necip Becit, Munacettin Ceviz, Hikmet Kocak

**Affiliations:** 1 Atatürk University, Erzurum, Turkey.

**Keywords:** Heart Ventricles/Surgery, Heart Aneurysm, Myocardial Revascularization, Coronary Artery Bypass, Cardiac Surgical Procedures/Methods

## Abstract

**Objective:**

The aim of this study was to evaluate early clinical outcomes and echocardiographic measurements of the left ventricle in patients who underwent left ventricular aneurysm repair using two different techniques associated to myocardial revascularization.

**Methods:**

Eighty-nine patients (74 males, 15 females; mean age 58±8.4 years; range: 41 to 80 years) underwent post-infarction left ventricular aneurysm repair and myocardial revascularization performed between 1996 and 2016. Ventricular reconstruction was performed using endoventricular circular patch plasty (Dor procedure) (n=48; group A) or linear repair technique (n=41; group B).

**Results:**

Multi-vessel disease in 55 (61.7%) and isolated left anterior descending (LAD) disease in 34 (38.2%) patients were identified. Five (5.6%) patients underwent aneurysmectomy alone, while the remaining 84 (94.3%) patients had aneurysmectomy with bypass. The mean number of grafts per patient was 2.1±1.2 with the Dor procedure and 2.9±1.3 with the linear repair technique. In-hospital mortality occurred in 4.1% and 7.3% in group A and group B, respectively (*P*>0.05).

**Conclusion:**

The results of our study demonstrate that post-infarction left ventricular aneurysm repair can be performed with both techniques with acceptable surgical risk and with satisfactory hemodynamic improvement.

**Table:** 

Abbreviations, acronyms & symbols	
CABG	= Coronary artery bypass grafting
CCS	= Canadian Cardiovascular Society
ECG	= Electrocardiography
EuroSCORE	= European System for Cardiac Operative Risk Evaluation
EVCPP	= Endoventricular circular patch plasty
IABP	= Intra-aortic balloon pump
INR	= International normalized ratio
LAD	= Left anterior descending
LIMA	= Left internal mammary artery
LV	= Left ventricle
LVEDD	= Left ventricular end-diastolic diameter
LVEDV	= Left ventricular end-diastolic volume
LVEF	= Left ventricular ejection fraction
LVESD	= Left ventricular end-systolic diameter
NYHA	= New York Heart Association

## INTRODUCTION

Other than very rare etiological causes, such as cardiomyopathy, trauma and syphilis, ventricle aneurysms occur following ischemic heart diseases, particularly transmural myocardial infarction. While they can occur immediately after acute myocardial infarction, they can also arise weeks or months later and are frequently seen in the anteroapical, apical and septal regions. Aneurysms occur in 3.5 to 38% of the cases due to insufficient replacement of necrotic myocardial tissue after acute transmural myocardial infarction with scar tissue in about six weeks^[1-4]^. Aneurysm disrupts the normal ellipsoid geometrical structure of the left ventricle (LV), leading to a dilated spherical ventricle with limited contractility and filling capacity^[5-7]^. The clinical presentation is usually congestive heart failure, *angina pectoris*, treatment-resistant ventricular arrhythmias, and arterial embolization^[8]^. The addition of coronary revascularization to the surgical treatment of the LV aneurysms improves the surgical outcome in these patients^[9,10]^.

The main goal of surgery in the LV aneurysms is to remove the scar tissue to maintain the normal filling volume and the geometric configuration of the ventricle, to prevent excessive filling in the diastole, and to remove the contractile paradox movement of the ventricle wall along the systole, thereby, fixing the function of the LV^[11,12]^.

The aim of this study was to compare surgical techniques in patients who underwent ventricular aneurysm repair and/or myocardial revascularization, and to evaluate early and late stage clinical outcomes of surgery with echocardiographic findings of the LV.

## METHODS

An approval was obtained from the local Ethics Committee for this study. A written informed consent was obtained from each patient. The study was conducted in accordance with the principles of the Declaration of Helsinki.

Surgical outcomes, pre- and postoperative clinical data and clinical outcomes at one year after the procedure, and echocardiography results of 89 patients who underwent post-infarction LV aneurysm repair and myocardial revascularization were retrospectively analyzed based on the inpatient clinical follow-up results. Patients underwent surgical techniques other than Dor procedure and linear repair were excluded. All patients were evaluated in terms of age, sex, angina intensity, functional capacity, LV ejection fraction (LVEF), number of coronary lesions, aneurysm location, aneurysm repair technique, comorbidities and morbidity and mortality rates. The outcomes of the applied ventricular aneurysm repair technique were statistically analyzed.

Coronary artery stenosis and anatomy were evaluated using coronary angiography and a coronary luminal stenosis ≥ 50% was considered significant, and bypass was performed. Left ventricular reconstruction was performed with endoventricular circular patch plasty (EVCPP) (Dor procedure) or linear repair technique. Pre- and postoperative 1-, 2-, 6-, and 12-month clinical outcomes of the patients (mean: 12.9±3.8 months), echocardiographic LVEF measurements, end-systolic and end-diastolic volumes and diameters were recorded. Based on the surgical technique used, Dor procedure patients were assigned to group A (n = 48), while those who underwent linear repair were assigned to group B (n = 41).

Angina intensity was based on the Canadian Cardiovascular Society (CCS) classification and functional capacity was based on the New York Heart Association (NYHA) classification.

All patients were operated under general anesthesia (fentanyl, midazolam, sodium thiopental, sevoflurane, lidocaine hydrochloride). In all patients, after induction of anesthesia, a triple-lumen venous catheter was placed into the internal jugular vein and electrocardiography (ECG) was performed; the patient was monitored for systemic and central venous pressure. Routine median sternotomy was performed in all patients. The left internal mammary artery (LIMA), the radial artery or saphenous vein graft were used. In patients who did not required an additional surgical procedure (*i.e*., valve replacement), aortocaval cannulation was performed. Cardiopulmonary bypass was performed, and cross-clamp was placed. To protect the myocardium, systemic and additional hypothermia was established with cold crystalloid potassium cardioplegia. Reperfusions were performed with cold blood cardioplegia every 20 min. Before opening the aortic clamp, reperfusion was performed with low potassium warm blood cardioplegia. The patient's body temperature was maintained between 28 and 32º C. Distal anastomoses of coronary lesions other than LIMA to left anterior descending (LAD) artery anastomoses were completed.

The decision on which technique to use in the repair was based on the size of the aneurysm during surgery and on the extent of the scar tissue. In the case of smaller lesions without a marked aneurysmal sac, linear repair was preferred, whereas endoaneurysmorrhaphy was performed in case of larger lesions with a marked neck and fibrotic sac.

**Fig. 1:**
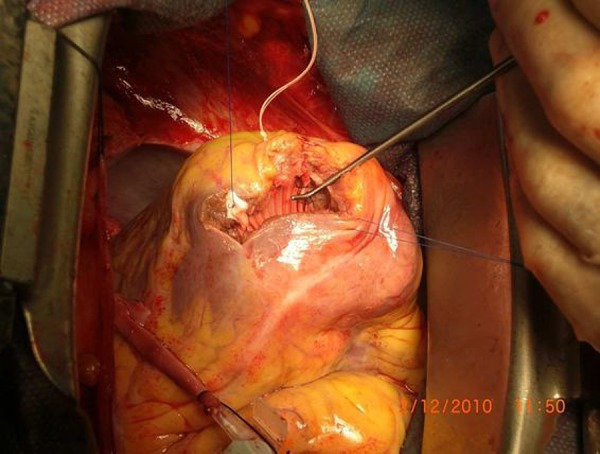
Operation image of the circular patch technique.

**Fig. 2:**
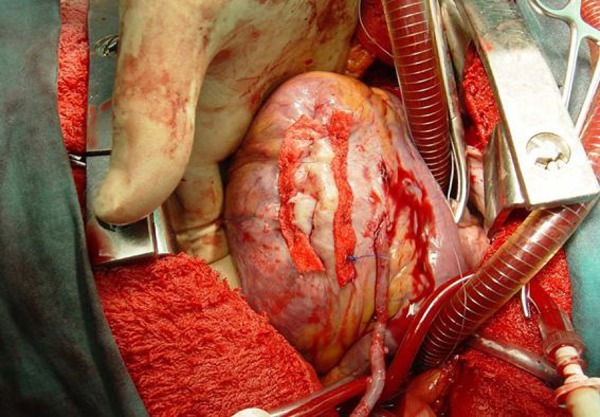
Operation image of the linear repair method.

In patients who underwent surgery with the Dor technique, a vertical incision was made in the anterior wall towards 2 to 3 cm lateral from the LAD artery to reach the aneurysmal sac. Once the aneurysm was opened and the aneurysm wall and, if present, the thrombus was removed, and a Dacron patch fitting to the alive and scar tissue margins was prepared. After the diastolic volume was measured with a balloon, the ventricular diastolic cavity was measured and the patch was implanted to the fibrous tissue at the border using 3/0 propylene continuous suture (Figure 1). The ventriculotomy margins on the ventricular wall were closed using two Teflon felts 1 to 2 cm wide and 6 to 7 cm long and with 2/0 propylene to retrace all layers. In patients who underwent surgery with linear repair technique, the aneurysmal sac was opened and the LAD artery was preserved and, then the thrombus was removed, if present. While paying attention not to reduce the ventricular cavity, the aneurysmal wall was resected to preserve the scar tissue (Figure 2). The aneurysmal tissue was closed with 2/0 Ethicon U stitches reinforced by two long Teflon patches. Mannequin balloon was used for measurement of the left ventricular volume in both groups. The balloon was inserted into the left ventricular cavity and the left ventricular balloon was inflated with normal saline as 40 cc/m^2^.

Based on the hemodynamics of each patient, medical treatment was prescribed. An intra-aortic balloon pump (IABP) was inserted in the patients with borderline ventricular functions. Once the patients woke up after about 3 to 6 hours, they were extubated on postoperative day 1 and were transferred to the ward on day 2. All patients routinely received acetylsalicylic acid 300 mg and warfarin 5 mg. Dose adjustment was made to maintain the International Normalized Ratio (INR) value between 2 and 2.5. The patients discharged on days 7 to 10 were scheduled for outpatient follow-up visits on day 10, at 1, 2, 6 and 12 months.

### Statistical Analysis

Statistical analysis was performed using SPSS version 20.0 software (IBM Corp., Armonk, NY, USA). Descriptive data were expressed as arithmetic mean and standard deviation. The independent Student's t-test was used to compare the variables between groups. The echocardiographic results were compared using the paired samples t-test. The Wilcoxon paired comparison test was used to compare the pre- and postoperative angina intensity and functional capacity. A *P* value of < 0.05 was considered statistically significant.

## RESULTS

Of the patients in group A, eight (16.7%) were female and 40 (83.3%) male, while in group B, seven (17.1%) were female and 34 (83%) male. The mean age of patients in group A was 59.6±7.3 years, while the mean age of patients in group B was 58.9±7.3 years.

Demographic and baseline clinical characteristics of the patients are shown in Table 1.

**Table 1:** Preoperative demographic characteristics of the patients.

Preoperative data	Group A (n=48)	Group B (n=41)	*P* value
Age (years)	59.6±7.3	58.9±7.3	0.39
Sex			
Male	40 (83.3%)	34 (83%)	0.015
Female	8 (16.7%)	7 (17.1%)	0.22
Hypertension	42 (87.5%)	36 (87.8%)	0.27
Diabetes mellitus	17 (35.4%)	11 (26.8%)	0.043
Renal function disorder	2 (4.16%)	2(4.87%)	0.89
Hyperlipidemia	34 (70.8%)	28 (68.3%)	0.83
COPD	23 (47.9%)	22 (53.2%)	0.91
PAH	12 (25%)	11 (26.8%)	0.36
Cigarette smoking	37 (77%)	30 (73%)	0.28
Thrombus in LV	10 (20.8%)	12 (29.3%)	0.16

COPD=chronic obstructive pulmonary disease; LV=left ventricle; PAH=pulmonary artery hypertension

The mean preoperative LVEF value was 38±6% (range: 27 to 54%). The mean LV end-diastolic diameter (LVEDD) and volume were 61.9±6.7 mm and 142.0±2.1 mL/m^2^, respectively. The mean LV end-systolic diameter (LVESD) and volume were 49.5±5.8 mm and 109.1±2.1 mL/m^2^, respectively. The mean preoperative CCS was 3.0±0.8, while the mean NYHA was 2.5±0.6. Total occlusion of LAD artery was observed in 61 (68%) patients. Based on the echocardiographic findings, 22 (25%) patients presented thrombus in the LV aneurysmal sac.

Aneurysm localization was apical in 70 (79%), apicoanterolateral in 13 (15%), anterolateral in three (3%), and posterobasal in two (2%) patients. Five (6%) patients had coronary lesion that did not require surgery. In addition, 39 (44%) patients presented a singular vascular lesion, while 45 (50%) had multiple vascular lesions.

The mean preoperative European System for Cardiac Operative Risk Evaluation (EuroSCORE) score was 5.6±2.4 (range: 1 to 16). The mean preoperative EuroSCORE was 5.2±4.0 in group A and 4.5±3.1 in group B. The primary indication for surgery was angina in 51 (57%) patients (CCS Class III-IV) and dyspnea in 44 (49%) (NYHA Functional Class III-IV). While the complaints of the patients in group A at the time of admission were NYHA (Class I-II) in 24 (50%) patients and NYHA (Class III-IV) in 24 (50%), these were NYHA (Class I-II) in 21 (50%) patients and NYHA (Class III-IV) in 20 (49%) in group B. Nine (19%) patients in group A and two (5%) in group B were in CCS Class I-II. However, 39 (81%) patients in group A and 39 (95%) in group B were in CCS Class III-IV.

Preoperative echocardiography revealed that 20 (42%) patients in group A and 11 (27%) in group B presented an ejection fraction < 35%. The mean values were 37.1±6.4% in group A and 40.3±6.4% in group B. The mean LVEDD was 62.7±7.0 mm in group A and 61.6±6.4 mm in group B, whereas mean LVESD was 49.6±6.2 mm in group A and 49.4±5.5 in group B. In addition, mean LVESV and left ventricular end-diastolic volume (LVEDV) in group A was 143±20.9 mL/m^2^ and 108.6±20.9 in group A, while in group B, these values were 140.8±21.9 and 109.7±22.9, respectively. Based on the echocardiographic findings of patients in group A, LV function was akinetic in 67% of the patients (n=32) and dyskinetic in 33% (n=16). In group B, these values were 51% (n=21) and 49% (n=20), respectively. The number of grafts per patient was 2.1±1.2 in group A and 2.9±1.3 in group B. Additionally, in group B, wrapping of the ascending aorta was performed in one patient, and mitral valve replacement was performed in another. In group A, mitral valve replacement was performed in two patients, septoplasty in one patient, atrial septal defect primary repair in one patient, and the Bentall procedure in two patients. The mean cross-clamp time was 74.8±29.1 min in group A and 62.8±19.6 min in group B. In addition, cross-clamping took more than 80 min in 18 (38%) patients in group A and seven (17%) in group B. The mean total bypass duration in group A was 118.9±48.1 min and 84.9±16.2 in group B. Total bypass took more than 120 min in 19 (40%) patients in group A and two (95%) in group B (Tables 2 and 3).

**Table 2:** Preoperative echocardiographic measurements.

Variable	Group A (n=48)	Group B (n=41)	*P* value
LVEF	37.1±6.4	40.3±6.4	0.39
LVEDD (mm)	62.7±7.0	61.6±6.4	0.27
LVESD (mm)	49.6±6.2	49.4±5.5	0.35
LVEDV (mL/m^2^)	143±20.9	140.8±21.9	0.29
LVESV (mL/m^2^)	108.6±20.9	109.7±22.9	0.16

LVEDD=left ventricular end-diastolic diameter; LVEDV=left ventricular end-diastolic volume; LVEF=left ventricular ejection fraction; LVESD=left ventricular end-systolic diameter

**Table 3:** Operative variables.

Variable	Group A (n=48)	Group B (n=41)	*P* value
No CABG	3 (6%)	2 (5%)	0.232
CABG 1	20 (42%)	7 (12%)	0.013
CABG ≥ 2	25 (52%)	32 (80%)	0.45
Cross-clamping time (mean± SD)	74.8±29.1	62.8±19.6	0.048
Cross-clamping > 80 min	18 (38%)	7 (17%)	0.03
TBS (mean)	118.9±48.1	849±16.2	0.016
TBS >120 min	19 (40%)	2 (95%)	0.002

CABG=coronary artery bypass grafting; TBS=total bypass time

The echocardiographic and clinical postoperative and one-year follow-up results of the patients were as follows: echocardiographic studies showed significant postoperative improvement in LV functions in both groups (*P*<0.05). The mean LVEF values were 41.0±4.6% in group A and 43.8±6.7% in group B (Table 4). In particular, LV systolic functions improved more in the patients who underwent Dor procedure. In the Dor procedure and linear repair groups, the mean preoperative NYHA classification were 2.5±0.6 and 2.5±0.5, respectively, and it increased to 1.8±0.7 and 1.9±0.5, respectively (*P*<0.001). The preoperative CCS classification were 3.12±0.84 and 3.21±0.85 respectively, and became 1.1±0.3 and 1.3±0.4 in the follow-up (*P*<0.001). In our clinical follow-ups, there was a significant improvement in angina symptoms and there were no differences between the groups (*P*>0.05). However, it was found that EVCPP more significantly improved patients' functional capacity (*P*<0.001). The mean postoperative LVEDD was 62.7±7.0 mm in group A and 61.6±6.4 in group B (*P*<0.001). The mean postoperative LVESD was 49.6±6.2 in group A and 49.4±5.5 in group B (*P*<0.001). The mean postoperative LVESV and LVEDV values were 86.6±21 and 50.0±9.8 mL/m^2^ in patients who underwent Dor procedure and 86.3±14.1 and 56.4±8.7 in the linear repair group, respectively (*P*<0.001).

**Table 4:** Preoperative echocardiographic and clinical results and one-year follow-up of patients.

Variable	Group A (n=48)			Group B (n=41)			*P* value
	Preoperative stage	Follow-up	*P* value	Preoperative stage	Follow-up	*P* value	
LVEF	37.1±6.4	41.0±4.6	< 0.001	40.3±6.4	43.8±6.7	< 0.001	0.03
LVEDD (mm)	62.7±7.0	54.9±6.1	< 0.001	61.6±6.4	56.4±5.4	< 0.001	0.39
LVESD (mm)	49.6±6.2	48.4±6.1	< 0.001	49.4±5.5	43.8±5.4	< 0.001	0.01
LVEDV (mL/m^2^)	143±20.9	86.6±21	< 0.001	140.8±21.9	86.3±14.1	< 0.001	0.96
LVESV (mL/m^2^)	108.6±20.9	50.0±9.8	< 0.001	109.7±22.9	56.4±8.7	< 0.001	0.001
NYHA class	2.5±0.6	1.8±0.7	< 0.001	2.5±0.5	1.9±0.5	< 0.001	0.32
CCS class	3.12±0.84	1.1±0.3	< 0.001	3.21±0.85	1.3±0.4	< 0.001	0.13

CCS=Canadian Cardiovascular Society; LVEDD=left ventricular end-diastolic diameter; LVEDV=left ventricular end-diastolic volume; LVEF=left ventricular ejection fraction; LVESD=left ventricular end-systolic diameter; NYHA=New York Heart Association

Postoperative medical treatment that was administered to our patients was similar to the preoperative treatment protocol. Accordingly, in the Dor procedure group and linear repair group, the rate of beta-blocker use was 80% (n=38) and 83% (n=34), respectively, the rate of Ca-channel blocker use was 31% (n=15) and 49% (n=20), respectively, the rate of angiotensin converting enzyme (ACE) use was 56% (n=27) and 44% (n=18), respectively, the rate of digitalis use was 21% (n=10) and 17% (n=7), respectively, and the rate of diuretic use was 41% (n=20) and 34% (n=14), respectively.

In the outpatient clinic follow-up of the patients, particularly in the Dor procedure group, it was found that digitalis and diuretic use decreased over time. Early in-hospital mortality was seen in one (2%) patient in group A and two (4%) in group B, due to low cardiac output. The mean preoperative LVEF was lower than 30% in the latter cases and, despite intense inotropic and IABP support in the postoperative period, both died due to LV failure on days 2 and 4 after surgery. During follow-up, one patient from each group died in the late stage. These patients were elderly and had multiple comorbidities. The total mortality rate was 5.6% (n=5). After surgery, six (12.5%) patients in group A and two (5%) in group B required IABP and inotropic support due to low cardiac output in the intensive care unit. Twelve (25%) patients in group A and 16 (39%) in group B developed postoperative arrhythmia. In case of use of IABP and postoperative arrhythmia were statistically significant (*P*=0.011, *P*=0.008, respectively). Five (10%) patients in group A and three (7%) in group B underwent revision due to hemorrhage. In the early postoperative period, 22 (46%) patients in group A and 15 (37%) in group B received inotropic support (dopamine). There was no statistically significant difference in the in-hospital mortality, inotropic support and revision surgery, and LV aneurysm repair techniques applied (*P*>0.05) (Table 5).

**Table 5:** Summary of postoperative early outcome.

Variable	Group A (n=48)	Group B (n=41)	*P* value
Hospital mortality	2 (4%)	3 (7%)	0.215
Inotropic requirement	22 (46%)	15 (37%)	0.380
Intra-aortic balloon pump	6 (12.5%)	2 (5%)	0.011
Re-exploration for bleeding	5 (10%)	3 (7%)	0.312
Postoperative arrhythmia	12 (25%)	16 (39%)	0.008
Acute renal failure	__	2 (5%)	0.45
Stay in ICU (days)			
1-3 days	43 (90%)	39 (95%)	0.445
≥4 days	5 (10%)	2 (5%)	

ICU=intensive care unit

Furthermore, two patients who had postoperative atrial fibrillation (one had chronic paroxysmal atrial fibrillation, and one had mitral valve replacement due to mitral insufficiency) were not eligible for intraoperative rhythm correction. Atrial fibrillation usually developed within the first week of surgery. These patients were referred for consultation to the cardiology department, and amiodarone infusion followed by oral treatment was initiated. Cardioversion was performed in two patients to restore normal sinus rhythm. Except one patient who underwent mitral valve replacement with linear repair, all patients were in sinus rhythm during follow-up, and amiodarone treatment was gradually discontinued. Hemodialysis was also performed in two (5%) patients due to acute kidney failure with borderline baseline creatinine values in the linear repair group. These patients did not require dialysis during follow-up. The mean length of stay in the intensive care unit was 2.7±1.7 (range: 1 to 13) days. In group A, 43 (90%) patients stayed for 1 to 3 days, while 10% stayed for 4 to 5 days. In group B, 39 (95%) patients stayed for 1 to 3 days, while two (5%) stayed for more than four days. In particular, advanced age and the presence of chronic obstructive pulmonary disease increased the length of stay in the intensive care unit. The patients in both groups were discharged after 7 or 10 days after the INR dose was adjusted.

## DISCUSSION

Left ventricular aneurysm formation is the most common mechanical complication of acute myocardial infarction^[13]^. These aneurysms involve all layers of the ventricular wall, have well-defined borders without ability of contraction, contain fibrotic and calcific tissues, are broad-based, contain 50% thrombus, and can be rarely ruptured^[4]^. The aneurysm can affect the neighboring normal myocardium and cause irregularities in papillary muscles, thereby causing mitral insufficiency. A mural thrombus is often found in the aneurysmal sac, however, the frequency of clinically detected systemic embolisms is low (2-5%)^[4,6]^. If aneurysmal tissue forms refractory, recurring, and life-threatening arrhythmias in cooperation with the reentry currents at its junction with the normal myocardium, surgical resection guided by electrophysiological mapping should be preferred^[10,14]^. In patients with symptomatic coronary artery disease, complete revascularization should be performed to allow the recovery of the neighboring myocardium after restoration of ventricular geometry^[9,15]^.

Clinical outcomes of LV aneurysms vary depending on the amount of myocardium affected, degree of ventricular distension, and accompanying coronary artery disease^[16]^. While 5-year survival rate with medical treatment is 8-12% in LV aneurysms, this rate can be increased from 75 to 90% with surgical treatment^[17]^.

Linear repair and Dor techniques, which are the two most frequently used techniques, have advantages and disadvantages. It is reported that EVCPP technique (Dor procedure) can be applied with low mortality (10%) in cases with akinetic myocardial scar, significantly improves LV function, restores the shape and function of the LV, and thus the outcomes of early and extended follow-up are satisfactory^[4,10]^. In the study by Shapira et al.^[18]^ in Dor procedure and linear repair technique, it was reported that although both had similar effects on the LV geometry, the Dor procedure caused a higher increase in the LVEF, improved long-term clinical recovery and improved functional capacity.

In the present study, we evaluated the early clinical results and echocardiographic measurements of the LV in patients who underwent LV aneurysm repair using two different techniques along with myocardial revascularization. Both groups were similar in terms of clinical characteristics, risk factors, indications for operation and additional procedures.

In the study by Chen et al.^[19]^, patient groups whose LV aneurysm repair was performed using two different techniques were analyzed. There was a significant improvement in the LVEF value in both groups. In the linear repair group, LVEF increased from 26.3±9% to 28.3±7.5%, and in the Dor technique group, the mean LVEF increased from 26.5±7.2% to 32.1±7.7%. Zheng et al.^[20]^ also reported that the mean postoperative EF value increased more in patients who underwent Dor procedure than patients who underwent linear repair. They suggested that this was because Dor procedure preserved the conical shape of the LV and caused a positive remodeling. In the study by Becit et al.^[7]^, the mean preoperative LVEF values in the Dor and linear repair groups were 0.30±0.06% and 0.31±0.07%, respectively, while during follow-up there was a significant improvement to 0.44±0.04% and 0.41±0.04%, respectively. In our patients, this value increased from 37.1±6.4% to 41.0±4.6% in the Dor group (group A), and from 0.3±6.4% to 43.8±6.7% in the linear repair group (group B). Echocardiographic studies also showed significant postoperative improvement in LV functions in both groups. In particular, systolic functions of the LV improved more in patients who underwent Dor procedure (*P*<0.05).

The surgical indications for LV aneurysms are *angina pectoris*, ventricular arrhythmias, dyspnea, and presence of systemic embolism^[4,15]^. In our study, the primary indication for operation was angina (CCS Class >II) in 88% of the patients, and dyspnea (NYHA functional Class ≥III) in 12% of the patients. Demirkilic et al.^[17]^ reported that the mean functional capacity in the preoperative period was 2.2. In another study, Ismailoglu et al.^[15]^ reported that the mean postoperative NYHA regressed from 2.19±0.75 to 1.23±0.63, and the CCS value regressed from 3.05±1.05 to 1.07±0.27. In the study by Tavakoli et al.^[6]^, the NYHA value decreased from 2.9±1.0 to 1.9±0.4 in the Dor group, whereas it decreased from 2.9±0.8 to 1.7±0.6 in the linear repair group. Similarly, in our patients, the mean preoperative NYHA value was 2.5±0.6 and regressed to 1.8±0.7 in group A and to 1.9±0.5 in group B (*P*<0.05). In addition, the CCS value of 3.0±0.8 regressed to 1.1±0.3 in group A and to 1.3±0.4 in group B (*P*<0.05). In all patients, a marked improvement in the postoperative functional capacity and anginal symptoms was detected. However, in the final visit, the NYHA functional capacity was improved in patients who underwent Dor procedure (*P*<0.05). Kesler et al.^[21]^ evaluated these two techniques in terms of the echocardiographic LV dimension and volume measurements and reported that there were no statistical differences between the outcomes of patch and linear aneurysm repair. In the study by Tavakoli et al.^[6]^, both LVEDV and LVESV decreased at a similar rate following surgery. In our study, the mean LVEDD decreased from 56.2±7.2 mm to 51±6.2 in group A and from 51.4±6.3 mm to 48.3±5.4 mm in group B, and LVESD decreased from 41.4±6.4 mm to 37.7±5.9 mm in group A and from 38.5±5.5 mm to 34.9±5.4 mm in group B. LVEDV regressed from 143±20.9 mL/m^2^ to 86.6±21 mL/m^2^ in group A, from 133.8±21.9 mL/m^2^ to 78.3±14 mL/m^2^ in group B, while the end systolic volume regressed from 100.4±21 mL/m^2^ to 58±1 mL/m^2^ in group A, from 92.7±22.9 mL/m^2^ to 53.4±8 mL/m^2^ in group B, and no significant difference was detected between the two groups.

Coronary artery bypass grafting (CABG) is also an important component of the LV aneurysm surgery, with a revascularization rate of 68 to 100% in the literature^[4,22]^. Although myocardial revascularization is controversial, many authors agree on the requirement of simultaneous CABG. With the operation in which aneurysmectomy and CABG are performed simultaneously, mortality decreased significantly and long-term survival increased. In the study by Mukaddirov et al.^[23]^, 35% of patients underwent CABG and the mean bypass number was reported as 1.3 grafts in each patient. In the study by Tavakoli et al.^[6]^, CABG was performed in addition to aneurysm repair in 84 patients (29 patients - 85% - in the Dor group and 55 patients - 90% - in the linear repair group). Erdil et al.^[9]^ also reported that the multiple vascular coronary artery disease rate accompanying LV aneurysm was high at 75%. In our study, there were 57 (64%) patients with multiple vascular lesions. We performed CABG in 94% of our patients with a mean graft number of 2.5±1.7. We suggest that complete coronary revascularization with aneurysm surgery can positively affect the long-term surgical outcomes and patients' quality of life. Furthermore, in the early postoperative period, only three (3.3%) patients were lost and only two (2.2%) died at one year of follow-up, and the absence of anginal pain in 93% supports our hypothesis. Zheng et al.^[20]^ also showed that the IABP insertion rate was 5.5% (n=8) in the Dor group and 4.8% (n=17) in the linear repair group, indicating no significant difference between the groups. In our patients, the IABP insertion rate was 12.5% (n=6) in group A and 5% (n=2) in group B.

Furthermore, independent determinant factors in the long-term survival were defined as LV function, age, unstable angina, and previous history of cardiac surgery by Carrel et al.^[24]^. Survival rates vary depending on parameters such as sex, presence of diabetes, type and severity of symptoms, location of the aneurysm, extent of coronary artery disease, and complete or incomplete revascularization. It has been known that advanced age, history of ventricular arrhythmia, triple vascular disease, weak LV formation and linear repair of the aneurysm decrease long-term survival rates^[10,15]^. In the study by Silveira Filho et al.^[25]^, the modified Dor procedure showed consistent LVEF improvements after long-term follow-up. Survival was comparable for all ventricular types and for the modified Dor and ventricular exclusion procedures. The EuroSCORE index is a useful index for the late survival assessment of ventricular restoration techniques. In our study, in the final visit, NYHA functional capacity was improved in patients who underwent Dor procedure (*P*<0.05).

## CONCLUSION

In conclusion, the results of our study show that LV aneurysm repair can be performed with low mortality and morbidity rates as an isolated CABG, yielding significant improvement in hemodynamic functions compared to medical treatment. In addition, post-infarction LV aneurysms can be repaired using both techniques with acceptable surgical risk and satisfactory early and late stage outcomes, particularly in LV systolic functions and functional capacity of the patient. These results also indicate that if LV restoration is performed particularly with complete coronary revascularization, regional afterload would reduce and ejection performance of the non-infarcted myocardium and cardiac functions would be improved with a significant recovery of the functional capacity.

**Table:** 

Authors' roles & responsibilities	
UK	Conception and design study; realization of operations and/or trials; analysis and/or data interpretation; statistical analysis; manuscript writing or critical review of its content; final manuscript approval
AÇ	Substantial contributions to the conception or design of the work; or the acquisition, analysis, or interpretation of data for the work; final manuscript approval
NB	Drafting the work or revising it critically for important intellectual content; final manuscript approval
MC	Drafting the work or revising it critically for important intellectual content; final manuscript approval
HK	Final manuscript approval

## References

[1] Evora PR, Tubino PV, Gali LG, Alves Junior L, Ferreira CA, Bassetto S (2014). A variant technique for the surgical treatment of left
ventricular aneurysms. Rev Bras Cir Cardiovasc Surg.

[2] Cooley DA, Walker WE, Moran JM, Michaels LL (1980). Surgical treatment of post infarction ventricular aneurysm:
evaluation of technique and results in 1533 patients. Surgery for the complications of myocardial infarction.

[3] Jones EL, Craver JM, Hurst JW, Bradford JA, Bone DK, Robinson PH (1981). Influence of left ventricular aneurysm on survival following the
coronary bypass operation. Ann Surg.

[4] Antunes PE, Silva R, Ferrão de Oliveira J, Antunes MJ (2005). Left ventricular aneurysms: early and long-term results of two
types of repair. Eur J Cardiothorac Surg.

[5] Lundblad R, Abdelnoor M, Svennevig JL (2004). Surgery for left ventricular aneurysm: early and late survival
after simple linear repair and endoventricular patch plasty. J Thorac Cardiovasc Surg.

[6] Tavakoli R, Bettex D, Weber A, Brunner H, Genoni M, Pretre R (2002). Repair of postinfarction dyskinetic LV aneurysm with either
linear or patch technique. Eur J Cardiothorac Surg.

[7] Becit N, Erkut B, Ceviz M, Ünlü Y, Koçak H (2007). Surgical treatment of postinfarction left ventricular aneurysms:
a comparison between patch and linear techniques. Turk Gogus Kalp Dama.

[8] Loop FD, Baue AE, Geha AS, Hammond GL, Laks H, Naunheim KS (1985). Aneurysms of the heart. Glenn's thoracic and cardiovascular surgery.

[9] Erdil N, Nisanoğlu V, Battaloğlu B, Cihan HB, Gülcan Ö, Ege E (2003). Early results of surgical treatment in patients with left
ventricular aneurysm. Turk Gogus Kalp Dama.

[10] Sartipy U, Albage A, Lindblom D (2005). The Dor procedure for left ventricular reconstruction. Ten-year
clinical experience. Eur J Cardiothorac Surg.

[11] Cooley DA (1989). Ventricular endoaneurysmorrhaphy: result of an improved method of
repair. Tex Heart Inst J.

[12] Krajcer Z, Elayda MA, Cuasay L (1992). Ventricular endoaneurysmorrhaphy: result of a new operation for
repairing left ventricular aneurysms in 100 patients. Tex Heart Inst J.

[13] Barratt-Boyes BG, White HD, Agnew TM, Pemberton JR, Wild CJ (1984). The results of surgical treatment of left ventricular aneurysm.
An assessment of the risk factors affecting early and late
mortality. J Thorac Cardiovasc Surg.

[14] Tonnessen T, Knudsen CW (2005). Surgical left ventricular remodeling in heart
failure. Eur J Heart Fail.

[15] İsmailoglu F, Özbaran M, Yüksel M, Buket S, Telli A, Durmaz İ (2002). Effectiveness of left ventricular aneurysm repairs and the
analysis of risk factors. Turk Gogus Kalp Dama.

[16] Fiore AC, Jatene AD, Baue AE, Geha AS, Hammond GL, Laks H, Naunheim KS (1996). Surgical treatment of left ventricular aneurysm. Glenn's thoracic and cardiovascular surgery.

[17] Demirkılıc U, Kuralay E, Yılmaz AT, Ozal E, Bingol H, Tatar H (1997). The effect of aneurysmectomy on operative mortality in
angiographically akinetic and dyskinetic left ventricular
aneurysms. Turk Gogus Kalp Dama.

[18] Shapira OM, Davidoff R, Hilkert RJ, Aldea GS, Fitzgerald CA, Shemin RJ (1997). Repair of left ventricular aneurysm: long-term results of linear
repair versus endoaneurysmorrhaphy. Ann Thorac Surg.

[19] Chen WY, Wu FY, Shih CC, Hai ST, Hsu CP (2009). Left ventricular aneurysm repair: a comparison of linear versus
patch remodeling. J Chin Med Assoc.

[20] Zheng Z, Fan H, Feng W, Zhang S, Yuan X, Wang L (2009). Surgery of left ventricular aneurysm: a propensity score-matched
study of outcomes following different repair techniques. Interact Cardiovasc Thorac Surg.

[21] Kesler KA, Fiore AC, Naunheim KS, Sharp TG, Mahomed Y, Zollinger TW (1992). Anterior wall left ventricular aneurysm repair. A comparison of
linear versus circular closure. J Thorac Cardiovasc Surg.

[22] Vicol C, Rupp G, Fischer S, Summer C, Dietrich Bolte H, Struck E (1998). Linear repair versus ventricular reconstruction for treatment of
left ventricular aneurysm: a 10-year experience. J Cardiovasc Surg.

[23] Mukaddirov M, Frapier JM, Demaria RG, Albat B (2008). Surgical treatment of postinfarction anterior left ventricular
aneurysms: linear vs. patch plasty repair. Interact Cardiovasc Thorac Surg.

[24] Carrel T, Metzger D, Jenni R, Turina M (1995). Early and late results of the surgical treatment of left
ventricular aneurysms: report of 105 patients. Schweiz Med Wochenschr.

[25] Silveira Filho LM, Petrucci O, Vilarinho KA, Baker RS, Garcia F, Oliveira PP (2011). A bovine pericardium rigid prosthesis for left ventricle
restoration: 12 years of follow-up. Rev Bras Cir Cardiovasc.

